# Remarks on the Employment of the Extract of Aconite in Rheumatism

**Published:** 1839-08-17

**Authors:** F. B. Watkins

**Affiliations:** Richmond, Va.


### MEDICAL EXAMINER.

DEVOTED TO MEDICINE, SURGERY, AND THE COLLATERAL SCIENCES.

No. 33.] PHILADELPHIA, SATURDAY, AUGUST 17, 1839. [Vol. II.

*Remarks on the employment of the Extract of Aconite in Rheumatism.* By F. B. Watkins, M. D.

Extract of aconite, though at one time (years ago) used, and now again revived by German physicians, is but little known or employed in this country. That it is a most valuable remedy in all rheumatic affections is well attested, and sustained by the experience of physicians in Germany and Sweden. I am at a loss to account for its being so little used, unless it be the imperfect, and consequently uncertain manner in which the extract is prepared.

My attention was directed to this subject, by observing a paper some time since in a number of the "American Journal of Medical Sciences." It is there so highly lauded by Professor Sigmond, and so respectable an array of physicians cited as commending it, that I sought the earliest opportunity of testing its efficacy. An occasion soon offered, and I now with pleasure accord my testimony in confirmation of its value.

I think I cannot more satisfactorily do this, than by giving an extract from my "case book."

The subject is a woman, aged thirty-six, in very indigent circumstances,—respectable,—has seen better days,—followed no particular employment.

I was called to her 28th May, 1839, and obtained from her this previous history:—About nine years ago Mrs. R. suffered from a long and tedious attack of intermittent fever; continued several months; soon after her recovery, had parotitis, then afterwards rubeola, and finally scarlatina; this succession of disease had nearly terminated her existence, and left her constitution very obnoxious to the action of morbific agents. About this time, a reverse of fortune compelled her to live in a damp, unhealthy situation; while in this condition, before the recuperative energies of her system could reinstate her in her previous healthy state, (for I should have mentioned she was hitherto of a strong, robust, and active constitution,) she had a very severe attack of acute rheumatism. From this she partially recovered; the disease was palliated, not eradicated. For the succeeding four years, the disease intermitted in character and severity. At one time, to use her own expression, she was quite smart,—at another, suffering intensely. During this period, had much medical advice; but, from some cause or other, was never entirely cured. Being in that condition of life in which quackery plays so powerfully on credulity, she submitted herself to its power. All her symptoms were aggravated; then, almost faithless, she submitted to specifics, nostrums, magic, trickery, incantations, *et id omne genus*, until her life, an existence of pain, suffering, and misery, seemed no longer desirable. Experiencing no relief from any quarter, she finally determined to *cut* the whole profession. For the last two years she has had no physician, except for some gastric disturbance, to which she is very subject. About twelve months since there appeared nodes on os frontis, which, in six weeks, suppurated and discharged, leaving now the cuticle adherent to the bone; had no other ulcerations. When first called, I observed incipient *nodes* on tibia of right leg. I was at first disposed to suspect a syphilitic taint; but a further acquaintance with the case, and her known reputable character, combined with other circumstances, soon dispelled such an opinion. These nodes (if they be such) I found to appear and disappear very suddenly and capriciously; I was afterwards induced to doubt their being legitimate *nodes*, and rather suppose them the result of an abnormal action of nervous influence, producing unequal, illegitimate contraction of muscular fibre. I may be mistaken: they, however, observed this capricious character; they were exceedingly painful. I here remark, that it is the right side that suffers mostly, but the left experiences less, merely by comparison; complains of much cephalalgia, with soreness of scalp; much swelling of right leg and foot during the day—subsides at night; pain very deep seated, as she says, "*in the bone.*" This is the previous history, and such her condition, when I sought the case, which, with the difficulty above detailed, I at last was *permitted* to treat.

*May 28th.*—I found her sitting up in a chair, almost unable to move, and never without assistance; rarely slept during the night; is constantly racked with pain; has not experienced one hour's immunity of suffering for more than three years; has not walked a quarter of a mile for that time. I will not impose on your indulgence by a detailed history of the treatment, collaterally, I adopted, but give as succinct an exposè as will be consistent with the intention of this communication.

Vin. semin. colchic. f℥j., three times per diem, afforded much relief, especially when combined with magnesia, or succeeded by ol. ricini.

*May 30th*—8 o'clock, A. M. Found her in bed; pulse eighty, feeble, compressible; tongue foul; colchicum has not operated. Ordered a dose of ol. ricin.

31*st.*—Much relieved; sitting up; cheerful; more comfortable; oil operated, with copious, offensive evacuations; slept well; no swelling on tibia; soreness, but no acute pain. Ordered pulv. ipecac. et opii gr. x., three times per diem; vin. semin. colchic. f℥ij., ibid. A manifest amendment followed this treatment, until the 9th of June, when an imprudent indulgence in cherries brought on a violent dysentery, which existed until the 15th, during which period there was a recurrence of rheumatic affection of great intensity. In this time I supposed the system entirely free of the influence of colchicum, and then determined to resort to the extract of aconite.

*June 15th*—7 P. M. Rheumatic symptoms very severe; dysentery cured. Ordered extract aconite gr. ¼, twice a day, gradually increased until gr. vi. p. d. should be taken, with pulv. ipecac. et opii gr. x. at night.

*17th.*—A decided amendment, which continued until the 3d of July, when I discharged the case, the patient having walked three miles the day before.

Whether the cure is to be attributed to the colchicum, or the aconite, or to a combination of both, is the question to be decided. I cite as authority, for the use of this remedy, G. G. Sigmond, Drs. Lombard, Stoerck, Rosenstein, Blom, Odhelius, &c. I will not presume to give the modus operandi,—since distinguished writers confess their inability to do so,—any farther than to say with them, that I believe its agency is through the medium of the nervous system. It would be out of place to give a history of this medicine; I refer to U. S. Dispensatory. My confidence, though not fully confirmed from the experience in *one* case, has been much increased by its remarkable efficacy in a case of gout, which came under my notice a short time since. An almost immediate amelioration and amendment of symptoms followed the first dose. Should you think this worthy of a place in your paper, and an opportunity occur soon for my testing still farther the value of this agent, I will with pleasure advise you of it.

N. B.—*August 8th.* I saw my patient this morning; she is well, and is most rapidly gaining flesh and strength, and says that she has not been so fleshy or strong for nine years.

*Richmond, Va.*, August 8th, 1839.

